# Evolution in Surgery

**Published:** 1895-06-01

**Authors:** 


### Evolution in Surgery.

In delivering the annual oration before the Medical Society of London, Mr. Pearce Gould did well to take for his subject the recent evolution of surgery, for in the history of the healing art no period has witnessed so great a change as has taken place within the past quarter of a century in the practice of surgery. It is not that there has been mere improvement in method, or that a higher excellence has been attained in manipulative dexterity; a complete change has taken place both in theory and in practice. Operative surgery not only now works in different fields from what it did in former times, but its very aims are different. The orator did not celebrate a progress which had been made along well-known lines, but proclaimed the attainment of a completely new platform from which fresh advance can be made in directions previously impossible—a step in the evolution of surgery. As Mr. Pearce Gould said, "The chief glory of this period lies in the almost entire transformation of surgical ideals that has occurred rather than in the improved methods of expressing them."

One great change that has come over surgery is the removal of the formerly admitted anatomical restrictions upon surgical operations. Thirty years ago the body was mapped out into areas in which operations might be performed, and others in which no operation was considered admissible. When every surgical procedure entailed grievous risk, and no means were known of certainly avoiding it, all surgeons felt the necessity of limiting operations to the irreducible minimum, but when they understood at last the true process of healing of uninfected wounds, such anatomical distinctions became meaningless, and the barrier to progress was removed. To-day we know that simple, well-executed surgical procedures are not in themselves productive of injury; the anatomical restrictions to operations have vanished, and the true limit to surgical possibilities lies now not in the anatomical structure of the part, but in its physiological importance to the organism. The surgeon no longer asks himself whether a structure is too delicate to be the field of his interference, but only whether he can operate upon it without injury to structures necessary to life, or without inflicting greater disabilities than those he wishes to relieve.

The next great change apparent in the surgical ideal is the higher regard in which the physiological integrity of the organism is now held. When surgeons were occupied with the question whether or no a wound would heal, which, even in days not so long ago, was always a matter of some uncertainty, the restoration of the physiological integrity of the parts involved was a mere refinement. If the wound healed the surgeon had done well, and then nature must do the rest. Now all is different. Where formerly surgeons spoke and thought merely of bringing the edges of a wound together, they now speak and think of the careful union of divided structures, so as to obtain the most perfect repair, using this word as meaning restoration of function; bones, nerves, and tendons are not now left in a jumble, to sort themselves; but, with serried ranks of sutures, each structure is held in its proper place, so that, when healed, its function shall not be interfered with.

The third idea which has found expression in the recent practice of surgery is a completely new conception of the personal responsibility of the operator. In days when surgeons saw nearly every wound suppurate, with erysipelas, pyæmia, and gangrene, as frequent accompaniments, and when they saw that neither their skill, nor the patient's sound health, nor the use of any known dressing, or indeed of none at all, was sufficient to guard against these appalling evils, their confidence in their art was sapped at its very foundation, and, what was worse, their confidence in their own power of determining the issue of their cases was destroyed. As a result surgeons were with grim irony called "brilliant" if only they could execute with dispatch and dexterity the feats of the operating theatre. If a large proportion of recoveries was obtained the man was apt to be called "lucky"; if failures predominated, again it was his "luck," shoulders were shrugged, the Deity was blamed, and the surgeon took comfort in his "brilliancy." To-day this is all as a dead language to us; the very slang of the hospital theatre is gone. Instead of brilliancy in execution only, we demand success; instead of speaking of "luck," we talk of surgical responsibility. Operations still fail, but instead of blaming the Deity we blame ourselves for the result. This is not merely progress, it is a fundamental change in our very conception of surgical work.

Again, while in former days surgeons removed diseases or their products, now they attack the very causes of disease, even entering upon healthy areas if by so doing they can influence the progress of a malady. Step by step Mr. Pearce Gould proved his point that the present position of surgery is not the result of a mere progression, however great, upon old lines, but is a true step in evolution. The whole oration took a high tone, and is calculated to give the public renewed confidence in the honour and conscientiousness of those to whom in times of peril, when surgery alone can save, they have to trust their lives.

